# Multi-Vendor Loyalty Programs: Influencing Customer Behavioral Loyalty?

**DOI:** 10.3389/fpsyg.2016.00204

**Published:** 2016-02-23

**Authors:** Teresa Villacé-Molinero, Pedro Reinares-Lara, Eva Reinares-Lara

**Affiliations:** Departamento de Economía de la Empresa, Universidad Rey Juan CarlosMadrid, Spain

**Keywords:** customer behavior, customer loyalty, behavioral loyalty, multi-vendor loyalty programs, reward programs

## Abstract

Loyalty programs are a consolidated marketing instrument whose adoption in many sectors has not been associated with appropriate comprehension of either their management elements or their effects. The purpose of this research is to contribute to knowledge about the effect of loyalty programs on repeat purchase behavior. More specifically, it seeks to discover whether joining a program changes the buying behavior of its members, and, if so, to study the profile of those whose behavior changes most. The intention was also to provide new study variables pertaining to multi-vendor loyalty programs, such as where they are joined or purchases in associated outlets as a result of behavioral loyalty. Research was carried out using a sample of 1200 individuals (31,746 purchases) belonging to a multi-vendor loyalty program. The study period was 13 years, 4 months, and split into two phases: before and after the joining the program. Different methodological approaches, such as the use of transactional databases that included pre-program-enrollment data and of the same sampling units throughout the study, were incorporated into the research with the aim of advancing academic knowledge regarding multi-vendor loyalty programs. Moreover, a type of program and market hardly dealt with in the relevant literature was analyzed. The results showed while the loyalty program had managed to reduce the time between purchases, it had not affected purchase volume or average expenditure. They also demonstrated the existence of a differential profile of customers who had changed their buying behavior to a greater extent. Finally, recency was identified as being the decisive variable in behavioral change.

## Introduction

Competition in today's market is intense, which makes acquiring new customers increasingly complicated and less profitable; hence the importance of securing the loyalty of existing customers. In this regard, Alet i Vilaginés and Nueno (2004) point to a shift in emphasis from gaining product trial to seeking customer loyalty, which, they argue, has become a fundamental strategic component for companies. Likewise, business strategies have gone from focusing on customer satisfaction to focusing on customer loyalty, primarily because of the understanding of the impact of loyalty on profits (Oliver, 1999). Some authors have suggested that customer loyalty is essential to achieving business profitability, as there is a high cost associated with acquiring new customers due to the low return on the initial transactions since, with many customers, profitability increases over the course of their relationship with a company (Anderson et al., 1994). Additionally, companies can benefit from other advantages related to having loyal customers: lower service costs, less price sensitivity, higher purchasing levels, and positive word-of-mouth (Sharp and Sharp, 1997).

The various existing conceptualizations of loyalty mainly revolve around two accepted dimensions (Bloemer and De Ruyter, 1998). The first is behavioral loyalty, which refers to commitment as expressed through behavior, that is, actual repeat purchasing. In this dimension of loyalty, importance is not given to the reasons for the repeat patronage, as what matters is the act of repetition itself. Thus, loyalty is measured in terms of the number of store visits, the number of repeat purchases at a single establishment, etc. The second dimension is attitudinal loyalty, which refers to the psychological or affective bond customers show toward a given product or brand in the form of a positive attitude toward repeat purchasing or even by engaging in positive word-of-mouth. Attitudinal loyalty shows the customers' preferences and inclination toward a given provider. Thus, customers are considered loyal when, in addition to engaging in repeat patronage with regard to a given product, brand, or establishment, they show an inclination, conviction, or favorable attitude toward it (Heiens and Pleshko, 1996), that is, when they evince both dimensions of loyalty. Given the importance of this subject, considerable research has been conducted on loyalty with a view to determining how to successfully build a long-term relationship with customers.

However, due to the difficulty of evaluating the psychological component of loyalty, most authors, while not denying the attitudinal dimension, have focused on the analysis of behavioral loyalty (O'Malley, 1998), which is easier to measure objectively.

In keeping with the business goal of achieving behavioral loyalty, recent years have witnessed a boom in loyalty programs based on cumulative reward point cards or obtaining discount vouchers (Zhang and Breugelmans, 2012). In order to encourage repeat purchases, these programs offer customers incentives (points or other exchange units), which can be redeemed for different items of value. The proliferation of loyalty programs reflects a changing market environment that is increasingly characterized by intense competition, more demanding and knowledgeable consumers, and a development toward relationship marketing and customer relationship management in marketing thinking and practice (Liu and Yang, 2009).

Loyalty programs, appropriately managed, are considered to allow structured and effective actions to manage, select, relate, and control customers' buying behavior.

Such programs have been extensively used by companies to motivate customers to increase their purchase volume and frequency. The assumption is that consumers will stay loyal only if suppliers provide an integrated service platform, that assures, that loyal customers are more favored than the rest of the market (Miranda and Kónya, 2008). It is thought, that correctly managed loyalty programs allow for structured, operational actions for managing, selecting, relating and controlling customer buying behavior (Banasiewicz, 2005).

McCall and Voorhees (2010) have argued, that the current lack of understanding of what factors drive a program's success is a major knowledge gap, that affects the optimization of how they are managed. Despite their development, the study of their effect on customer behavior in existing literature has provided contradictory results (Bojei et al., 2013). Dorotic et al. (2012), in their descriptive review approach to this issue, explained this disparity by claiming that the differences in results depended on several factors: (a) the area of application, or rather, the products involved or the markets where the programs were launched, (b) the type of customer segments analyzed, (c) the type of program (mono-sponsor/multi-sponsor), and (d) the methodology used in the research. These authors highlighted the need to continue investigating loyalty programs on the basis of the factors identified which affect the results.

This article will focus on the impact of loyalty programs on repeat purchase behavior (purchase loyalty). This is because, in practice, only repeat purchase behavior is rewarded, and not attitude, as loyalty schemes are often based on classic promotional techniques, with delayed, or immediate rewards (gifts, price reductions, points, etc.) or relationship marketing techniques (access to privileges or services, special status, individualization, etc.), which encourage consumers to purchase more often and remain loyal to the store. We can thus assume, that if a certain number of loyalty cardholders make similar changes in their buying behavior, there will be visible changes in repeat purchase patterns at a store level (market share, penetration, average purchase frequency; Meyer-Waarden and Benavent, 2006).

This research is focused on studying behavioral loyalty in multi-vendor programs. These consist of companies from different sectors joining together under one umbrella brand, sharing the implementation and management costs of the program, with the aim of providing cardholders with a diversified offer of where to obtain and redeem points rapidly. A stand-alone program itself does not promote the acquisition of new customers as easily as a multi-vendor loyalty program, by which new customers can be won over from other program participants (Rese et al., 2013). According to authors such as Lemon and Wangenheim (2009), this type of program has strategic advantages due to inter-company cooperation favoring cross-selling. Nevertheless, others (Sharp and Sharp, 1997; Dorotic et al., 2011) limited the networking effects across competing partners.

Within this context, the proposed general aim was to study whether joining a multi-vendor loyalty program caused a change in buying behavior, although several, more specific objectives were also set. Firstly, the aim was to analyze whether, in those cases where a change in buying behavior did occur after the program was joined, the customers who most modified their behavior had a differentiable profile. If so, companies would be able to carry out relationship marketing activities aimed at this specific group. And secondly, the intention was to deepen the study of multi-vendor loyalty programs in order to examine the possibility of extrapolating the existing knowledge about mono-sponsor loyalty programs toward them. Thus, what were effectively new study variables when related with buying behavior in the field of multi-vendor programs were used. These included: the place of enrolment, purchases in other establishments belonging to the program, the number of other establishments where the customer buys using the program, and even the feasibility of using variables such as recency or accreditation level for the operational management of multi-vendor programs.

The field of application for this study was small businesses; more specifically, medium-priced consumer goods, such as those found at optical shops, were analyzed. This sector application represents an advance in the business management of these programs since, it does not deal with a sector, that has been routinely studied (such as airlines, hotels, and grocery stores).

Finally, for the purposes of making advances in loyalty program research with adequate scientific rigor, a new methodology was applied, consisting of the use of transactional databases incorporating purchases made prior to when the program was joined and sampling units, that remained constant throughout the study (longitudinal analysis).

## Conceptual framework

### The influence on behavioral loyalty of an establishment joining a multi-vendor loyalty program

Authors such as Lewis (2004), Taylor and Neslin (2005), Kivetz et al. (2006), and Bridson et al. (2008) have stated, that loyalty programs permit the modification of customer buying behavior and, therefore, improve loyalty to the establishment in question. Drèze and Hoch (1998) claimed, that such programs had a positive effect on both the size and the average cost of purchases. Likewise, for the vast majority of researchers, belonging to a program reduced the time between purchases (Lewis, 2004).

Other authors have also confirmed the influence of loyalty programs on behavioral loyalty, but limiting their effects to the short term, therefore, usually associating them with promotional rather than relationship marketing activities (Benavent and Crié, 2000).

However, Dowling and Uncles (1997) and García Gómez et al. (2006) put further limits on the influence of loyalty programs to change customer buying behavior. Sharp and Sharp (1997) even alleged, that such programs neither modified buying behavior nor increased market share. Similarly, Long and Schiffman (2000) maintained that only a small number of customers modified their behavior after joining a program.

Finally, Meyer-Waarden (2008) pointed out, that changing buying behavior in multi-vendor programs was even more complicated than in mono-sponsor ones. In this type of program, in order to obtain their reward, customers do not even need to buy more frequently in outlets, that they did not use before, since, by being multi-vendor, they can continue accumulating points in their usual outlet. Moore and Sekhon (2005) confirmed the lack of influence of multi-vendor programs on improving market share.

This rather contradictory background and the methodological limitations identified in previous research make the formulation of the following hypothesis coherent:

H1: The incorporation of an establishment into a multi-vendor loyalty program produces a change in the behavioral loyalty of its customers, as measured in terms of buying behavior.

In this research, in accordance with previous literature, buying behavior was measured on the basis of the following variables:

- Volume of annual basket (Verhoef, 2003; Lewis, 2004; Liu, 2007; Lacey, 2009).
- Average purchase price (García Gómez et al., 2006; Meyer-Waarden, 2008; Smith and Sparks, 2009; Ponzoa and Reinares, 2010).
- Average inter-purchase time (Lewis, 2004).
- Number of items and categories per purchase (Smith and Sparks, 2009).
- Annual number of visits to the store (Sharp and Sharp, 1997; Benavent and Crié, 2000; Lewis, 2004; Smith and Sparks, 2009).
- Average annual expenditure (Meyer-Waarden, 2008; Smith and Sparks, 2009).

### The profile of those customers who most modified their buying behavior after joining the program

According to various authors, actions carried out within loyalty programs do not have the same impact on response for all customers (Dorotic et al., 2012). For example, loyalty programs apparently have more effect on the “light” and “moderate buyers” (Lal and Bell, 2003). This, of course, seems logical since the “heavy users” have less capacity to increase their purchasing volume or frequency; nevertheless, they maintain their high usage levels. Moreover, “early adopters” are usually heavy users, given that they are already loyal customers, living close to the shop, holding a joint family card and with a tendency to join up quickly in order to take advantage of the program's benefits (Leenheer et al., 2007; Demoulin and Zidda, 2009). Therefore, since differences in buying behavior due to customer type exist, it can be stated that one of the main advantages of loyalty programs is that they make relationship segmentation possible (Reinares and Ponzoa, 2008; Drèze and Nunes, 2009).

Within this context, the intention was to analyze the buying profile of the customers who most change their behavior, thus allowing for the identification of those customer groups that it was more interesting to incorporate into the program, due to their more favorable response.

With the aim of checking this argument within the realms of multi-vendor programs, a new hypothesis was formulated. Therefore, for each one of the buying behavior variables used to measure behavioral loyalty to the program, the group of customers who most modified their buying behavior after joining the program was selected. This group consisted of 5% of the total. The objective was to compare their behavior with, that of the rest in order to verify whether there existed differences between the two groups. The proposed hypothesis was as follows:

H2: Significant differences exist between those customers who change their buying behavior to the greatest extent after joining a program and the rest.

### The influence of the point accumulation rate on behavioral loyalty

The point accumulation rate is considered to be a very useful variable in detecting high-value customers. It is defined by the number of points that a customer builds up using the program's card for purchases within a given period of time (Ponzoa and Reinares, 2010). Loyalty programs use this variable to classify customers on the basis of their program transactions, or rather, the higher the accumulation rate, the greater the number of purchases. Therefore, the intention was to verify whether, in effect, the accumulation rate had a bearing on the traditional buying behavior variables for multi-vendor programs.

In a multi-vendor program, the accumulation rate depends on the total expenditure within the program, which is to say, within the whole set of establishments pertaining to it. Therefore, it is more easily increased due to the greater number of outlets where points can be accumulated. At the same time, however, it is more difficult to use the accumulation rate to identify those customers with higher transactional volume (since they may be very active in some outlets while being completely inactive in others).

This can be attributed to the “double jeopardy phenomenon”: that is to say, the greater the market penetration of the brand, the greater the buying frequency and the category quota bought, and vice-versa, so loyalty programs are more effective for market leaders than for small companies (McGahan and Ghemawat, 1994). Smaller companies not only have fewer customers, they also have a lower purchase frequency (Ehrenberg et al., 1990). Therefore, companies belonging to a loyalty program can obtain very varied results, as previously described, depending on the market penetration of each one's brand.

Thus, with the aim of testing whether this variable could be useful in the taking of operational decisions in the businesses belonging to multi-vendor programs, the following hypothesis was formulated.

H3: The accreditation level (point accumulation rate) of customers in a multi-vendor loyalty program influences behavioral loyalty toward one of the member establishments.

### The influence on behavioral loyalty of the establishment where the program is joined

Multi-vendor loyalty programs exhibit differentiating elements, with respect to mono-sponsor ones, which it is necessary to analyze. The possibility of accumulating and redeeming points in the different member establishments of the multi-vendor platform makes a deeper study of this differential aspect necessary. Thus, questions as to whether the establishment in which the program is joined exerts an influence on behavioral loyalty or whether purchasing in other member establishments implies a specific buying behavior arise.

If prior related literature is analyzed, according to Meyer-Waarden (2008), the programs first attract the high-volume shoppers of the establishment in which the multi-vendor program is joined. This is due to the so-called “self-selection” effect by which the “heavy users” show a greater probability of participating in a program than other customer types with a lower buying volume and lesser frequency (Leenheer et al., 2007). Therefore, by extrapolating these results to the multi-vendor loyalty program environment, one can logically infer that the customers that joined the program through the analyzed retailer (and not through another of the program's member establishments) could well have a buying behavior similar to that of the “heavy users” with regard to the purchase of products in that particular retailer since they were already buying at that particular optical shop prior to its implementing a multi-vendor loyalty program. Thus, these customers would have been “self-selecting” themselves. On the other hand, those customers who joined the multi-vendor program through other affiliated companies (establishments from different, non-competing sectors: fuels, food, banking, etc.) would behave in a similar way to “light users” in the purchase of optical products (less frequent, lower volume), but would exhibit a greater change in buying behavior.

So, taking this into account, the following hypothesis regarding the establishment where enrollment takes place was proposed:

H4: The establishment where a program is joined has an influence on the change in buying behavior after joining a multi-vendor loyalty program.

### The effect on behavioral loyalty of buying in other multi-vendor program member establishments

There have been very few studies on the impact, that the associate companies in a multi-vendor loyalty program have on buying behavior, since the majority of studies have been focused on mono-sponsor programs where relationships with “partners” simply do not exist.

Among those who have analyzed multi-vendor programs, Dorotic et al. (2011) compared the response of multi-vendor loyalty program members to individual brand promotions with their response to joint promotions within the program itself. The results demonstrated the low effectiveness of joint promotions once customers had joined the program, in such a way, that the customers responded better to the individual promotions of each brand. Therefore, within the realm of promotions, multi-vendor programs had not displayed any advantage over mono-sponsor ones.

Given the scarcity of bibliographical sources specifically relating to multi-vendor programs, prior literature regarding mono-sponsor programs with associated companies was also analyzed and taken into account, since these are the programs that most resemble multi-vendor ones.

In this area, authors such as Lemon and Wangenheim (2009) studied how the use of and satisfaction with buying the principal product of the program (“core service”) influenced the purchase of other products in establishments associated with it.

They specifically verified how, as use of and satisfaction with the core service increased, so did cross-purchasing from other services in the program, and vice versa. However, they confirmed that cross-purchasing in one category did not seem to strengthen cross-buying between other categories; that is to say that the relationship was only confirmed when there existed a main product/core service within the program. Moreover, according to these authors, this relationship was influenced by the kind of service that the partners in the program offered; the more, that this fit with and complemented the core service, the greater was the cross-purchasing effect.

In contrast with these results, other authors have found, that satisfaction with the core service of an enterprise did not exert a positive influence on the cross-purchase of other services, and also, that this itself was negatively influenced by the number of services joined to it in the recent past (Verhoef, 2003). The problem with adapting these results to multi-vendor programs is the lack of a “core service” or central product. Nevertheless, according to Moore and Sekhon (2005), the majority of the purchases of a multi-vendor program customer take place in the two leading member establishments, which act as the driving force of the program.

Thus, it would be interesting to know whether the customers that only shopped at the optical shop (as if the program were mono-sponsor) demonstrated higher behavioral variable values (shopping basket, average purchase price, etc.) than the customers that also bought in other member companies (food, clothing, etc.). Therefore, the following hypotheses were proposed:

H5a: Purchasing in other multi-vendor program member establishments influences behavioral loyalty toward other companies associated with the program (in this case: the optical shop).
H5b: The number of different sectors in the program in which a customer buys using the multi-vendor program influences behavioral loyalty toward other companies (the optical shop) associated with the program.

## Method

As explained in the presentation of the dimensions of loyalty, like other studies (Nilsson and Olsen, 1995), this study focuses on the behavioral dimension. Several authors (Lewis, 2004; Lacey, 2009; Lemon and Wangenheim, 2009) considered that, in order to measure the success of a program, it was necessary to carry out longitudinal research, which observes how buying behavior changes over time, as opposed to cross-sectional studies, which offer a snapshot view of buyer behavior. Similarly, the longitudinal research carried out in this field has normally analyzed either the change in buying behavior after joining the program (Lal and Bell, 2003; Verhoef, 2003; Taylor and Neslin, 2005; Liu, 2007; Tsao et al., 2009), or the change produced by changing from a traditional program to one with specific characteristics (Zhang and Breugelmans, 2012).

With the aim of overcoming the limitations identified in other work, the proposal in this study was to compare consumers' behavior before and after they joined the program.

Of the two multi-vendor loyalty programs existing in the Spanish market, the one that possessed the most ideal formal characteristics for fulfilling the proposed objectives and the greatest typological representativeness of Spanish households was chosen for the transactional data analysis.

The target population consisted of Spanish residents, aged 18 and over, who were loyalty program members. According to data from the company PSM (2013) the study universe consisted of some 22,750,000 individuals.

The study took place in 14 urban areas in Spain and its field of application comprised small retail establishments (more specifically, optical shops). The study period was 13 years 4 months, giving it a greater timespan than previous research. The study period was divided into two parts: (a) “before joining the program” (January 2, 1996–March 30, 2002) and (b) “after joining the program” (April 1, 2002–May 9, 2009). The date the establishment joined the program was April 1, 2002.

The sampling frame comprised those customers of the optical shop chain who, being members of the selected loyalty program, had made at least one purchase in each of the two periods under study. The selection procedure for the sampling units within the program used was simple random sampling.

A sample of 1,200 individuals who had effected a total of 31,746 purchasing actions in the two periods studied was obtained.

Concerning the information about the ethics requirements, this study was made according to criteria of the Ethics Department of the Rey Juan Carlos University and to the ICC/ESOMAR International Code on Social Research. Personal or sensitive data were deleted of the initial database because they did not provide relevant information for the analysis.

The study variables were calculated by cross-referencing the electronic transactional data from the multi-vendor program with that of the retailer studied.

The initial database was modified with a complex conversion process to suit the objectives of the study, and some existing variables were transformed according to the criteria used in the literature; to calculate these variables, algorithms programmed in *Visual Basic for Applications* were used. Also, specific new variables on the operation of loyalty programs were added based on the recommendations given by industry experts.

Finally, a total of 45 variables grouped into 9 constructs were used for the analysis, related to: (a) purchase behavior, (b) change in buying behavior after joining the program, (c) purchase behavior by category of product purchased, (d) recency, (e) multi-vendor purchase behavior (variables related to purchases made in other establishments), (f) type of consumer, (g) level of accreditation, (h) communication channels used by the multi-vendor program, and (i) redemption of points.

## Results

In order to verify the indicated hypotheses, a *T*-test for related samples and, later, a Wilcoxon test to confirm the results obtained were carried out (see Table 1).

**Table 1:** **Results: T and Wilcoxon tests**.

	***T***	***p***	***W***	***P***	***Df***
Volume of basket	−0.20	0.85	712,952	0.68	2389.8
Average purchase price	6.5	1.00e−10	840,639	1.18e−12	2159.1
Inter-purchase time	3.57	0.00	775,892	0.00	2384.5
Average number of visits	−3.61	0.00	662,643	0.00	2383.6
Average number of articles	7.81	8.312e−15	851,466	5925e−15	2330.1
Average number of categories	3.8	0.00	793,922	1007e−05	2369.3
Average annual expenditure	2.43	0.01	752,230	0.06	2368.6

The first variable used to measure the influence of joining the program on behavioral loyalty was volume of basket. The average per customer increased after joining the loyalty program, but, as can be seen in Table 1, this increase was not statistically significant since in neither of the two tests was the significance level obtained lower than 0.05.

The second variable used for the same ends was the average purchase price. This value was very slightly lower in the time after joining the program in comparison with that spent in the preceding period. The results confirmed that the difference in averages was significant. This decrease was due to a progressive price reduction that some products at the optical shop had been undergoing and not to the program in itself.

The inter-purchase time was also reduced upon joining the program. The tests used confirmed, that these differences in average were indeed highly significant since the critical levels were almost 0. This variation was very positive for the establishment, as hoped for, since it implied a greater buying frequency. Likewise, and as a result of this change, it was noted that joining the program had increased the average number of visits per year to the member establishment. The tests used to check the difference in averages confirmed, that this difference was also significant.

As happened with the average purchase price, the change in the number of articles and product categories purchased was not as expected since these decreased after the program was joined. This difference in averages was confirmed statistically as can be seen in Table 1.

The last measurement variable used for the purposes of this research was the average annual expenditure of the customers. As was the case with the average purchase price, there was a decrease in the second period analyzed. However, here, the two tests used differed. In the *T*-test the results proved to be significant (*p* = 0.01), while in the Wilcoxon test they were not (*p* = 0.06); the significance level was slightly higher than the critical value. It could therefore not be confirmed that joining the program had caused a change in average annual expenditure.

These results partially confirmed Hypothesis 1. Although a change took place in all the variables used, after joining the program, the differences only turned out to be significant and positive for the establishment in the case of reducing inter-purchase time and increasing the average number of shop visits.

Next, in order to analyze the profile of those customers who had most changed their buying behavior after joining the program, the sample was split into two groups comprised of those customers who had greatly changed their behavior compared to those who had done so to a much lesser degree. The difference in averages considered was the difference between the 5% of customers that had most increased their buying behavior and the remaining 95% who had done so to a much lesser extent. Afterwards, a comparison of the differences in behavior for the two sub-samples both “before” and “after” was carried out for each of the buying behavior variables.

The results obtained for each one of the buying behavior variables demonstrated, that the change produced was related to several variables from the period prior to when the loyalty program was joined. Thus, the variables, that had most significantly influenced the buying behavior of the customer group analyzed were: recency in the pre-joining period and previous average expenditure on key optical products, lenses and graduated lenses (see Table 2). More specifically, recency was significant for all the buying behavior variables. Those customers who exhibited greater recency before joining were identified as those who most changed their volume of basket (202.14 days since, the last purchase), their average purchase price (recency: 417.20 euros), their average inter-purchase time (recency: 375.66 days), the average number of articles bought (recency: 289.74 days), and their average number of store visits (recency: 211.69) and also those who most increased their average annual expenditure (120.87 euros).

**Table 2:** **Profile analysis of the greatest buying behavior modifiers (5%)**.

**Buying behavior variables**	**Most influential buying behavior change variables**			
	**MA**	***T***	***p***	***Df***
**RECENCY BEFORE JOINING**				
Volume of basket	202.14	−4.56	0.00	75.88
Average purchase price	417.20	−5.12	0.00	63.30
Inter-purchase time	375.66	−4.58	0.00	63.27
Average number of articles	289.74	−3.44	0.00	62.00
Average number of visits	211.69	−4.67	0.00	76.25
Average annual expenditure	120.87	−2.07	0.04	68.47
**AVERAGE EXPENDITURE ON CORE SERVICE/PRODUCT BEFORE JOINING**				
Volume of basket	25.81	−4.75	0.00	116.22
Average purchase price	34.33	−2.41	0.02	64.13
Inter-purchase time	30.66	−2.10	0.04	63.83
Average number of visits	23.45	4.54	0.00	129.31
Average annual expenditure	24.74	−2.34	0.02	69.16
**ENROLLMENT IN THE ANALYZED ESTABLISHMENT (OPTICAL SHOP)**				
Average number of (store) visits	−0.14	2.23	0.03	66.36
**PURCHASE IN THE FUEL SECTOR USING THE PROGRAM**				
Average purchase price (basket)	0.016	−2.41	0.02	65.44
**PURCHASE IN THE CLOTHING SECTOR USING THE PROGRAM**				
Average number of (store) visits	0.14	−2.42	0.02	62.66

In the case of average annual expenditure on core products (lenses and graduated lenses), this greater change was noted for all the buying behavior variables except that of the average number of articles purchased.

It is also interesting to note, that the consumers who showed the greatest change in number of store visits (-0.14) were customers whose enrollment had taken place in program member establishments other than the optical shop in question and who bought in other sectors, such as clothing (0.14). Moreover, purchasing in the fuel sector caused a greater increase in the average purchase price for the customers in the analyzed group (0.016). Therefore, on the basis of these results, Hypothesis 2 (H2) can be accepted.

Having determined the influence of recency on buying behavior change, the influence of the point accumulation rate on behavioral loyalty was then verified. In order to do this, the degree of relationship between the average accumulation rate and each one of the buying behavior variables in the post-joining time period was studied (see Table 3). The results of the Pearson correlation coefficient showed a negative relationship between the average accumulation rate and the following variables: volume of basket, average number of articles bought and number of store visits. The relationship was therefore positive for the remaining variables—average purchase price, inter-purchase time, average number of product categories bought and average annual expenditure. The correlation coefficients and *p*-values confirmed the absence of correlation between the analyzed variables. Therefore, the third hypothesis was rejected.

**Table 3:** **Correlation analysis between average accreditation rate and behavioral loyalty: Pearson's correlation coefficient**.

	***R***	***T***	***p***
Volume of basket	−0.01	−0.50	0.62
Average purchase price	0.02	0.86	0.39
Inter-purchase time	0.01	0.32	0.75
Average number of articles bought	−0.01	−0.35	0.72
Average number of categories bought	0.00	0.09	0.93
Average number of visits to the store	−0.01	−0.30	0.76
Average annual expenditure	0.02	0.54	0.59

*(N = 1200)*.

Finally, the results obtained for the variables particular to multi-vendor programs—the establishment where enrollment occurred and purchases in other program member establishments—were analyzed.

First, the influence of the “enrollment establishment” on buying behavior change after the multi-vendor program was joined was examined. The results of the tests carried out confirmed, that the differences, that existed on the basis of the enrollment establishment were only significant with regard to the variables (see Table 4): (a) average number of categories bought on each visit, which was higher for customers who joined at the optical store [*T*_(1, 1095)_ = −2.79, *p* = 0.01, and *W*_(1, 1095)_ = 162.940, *p* = 0.02]; and (b) average number of visits, which was higher for those who joined at other establishments. Here it should be pointed out that the results obtained with the *T*-test [*T*_(1, 1186.9)_ = 1.71, *p* = 0.09] were not significant, whereas with the non-parametric contrast of the Wilcoxon test it was confirmed that they in fact were [*W*_(1, 1186.9)_ = 188, 004, *p* = 0.05], although with only the minimum critical level. Therefore, Hypothesis 4 was partially rejected.

**Table 4:** **Analysis of the influence on buying behavior change of the establishment where enrollment took place**.

**Variable**	**M**		***T***	***p***	***W***	***P***	***Df***
	**Other (*N* = 684)**	**Optical shop (*N* = 516)**					
ΔVolume of basket	0.13	0.06	0.49	0.63	182,016	0.35	1197.8
ΔAverage purchase price	−8.88	11.76	−1.58	0.11	166,703	0.10	1033.6
ΔInter-purchase time	−57.61	−30.85	−1.17	0.24	166,609	0.09	1190.4
ΔAverage number of articles	−0.28	−0.14	−2.12	0.19	165,843	0.07	1107.8
ΔAverage num. of categories	−0.09	0.00	−2.79	0.01^*^	162,940	0.02^*^	1095.0
ΔAverage number of visits	0.26	0.14	1.71	0.09	188,004	0.05^*^	1186.9
ΔAverage annual expenditure	−7.33	−0.23	−0.73	0.03^*^	177,022	0.07	11,721.0

* *p < 0.05*.

With respect to the effect on behavioral loyalty of buying at other multi-vendor program member establishments, it was verified, that the customers who purchased at other program establishments, and not only at the optical shop, had greater basket volumes, a higher average purchase price, a lower inter-purchase time, and therefore, a higher average number of visits to the store, and, finally, a greater average annual expenditure. On the other hand, those customers who used the program as if it were mono-sponsor, only using the loyalty program when buying at the optical shop, did so on average with a higher number of articles and categories. However, these differences between the two groups of customers were only found to be significant with respect to the average annual expenditure variable [*T*_(1, 399.84)_ = −2.0, *p* = 0.05 *y W*_(1, 399.84)_ = 103, 437, *p* = 0.05). Thus, Hypothesis 5a was partially rejected.

In order to analyze the influence on behavioral loyalty of the number of other sectors of the program in which a customer purchases using the multi-vendor program, the Kruskal–Wallis *H*-test was used (see Table 5). The results showed that customers who only bought at the optical shop had a higher average number of articles bought on each visit to the store, while those who also bought in another sector of the program had a bigger basket volume and number of visits and, therefore, also a lower inter-purchase time. Moreover, those customers who bought in more than two different sectors within the program exhibited a higher average purchase price and also a higher average annual expenditure. However, the *p*-values of the Kruskal–Wallis H-test indicated that the differences found on the basis of the number of program sectors with purchases were not significant since they all substantially exceeded the 0.5 level. Thus Hypothesis 5b was also rejected.

**Table 5:** **Analysis of the influence on behavioral loyalty of the number of other sectors where purchases took place: Kruskal–Wallis *H*-test**.

**Variable**	***M***			***H***	***p***	***Df***
	**0**	**1**	**2 or more**			
Volume of basket	2.58	2.87	2.68	0.4554	0.80	2
Average purchase price	148.31	156.74	179.52	1.5613	0.46	2
Inter-purchase time	558.45	514.46	613.33	2.1845	0.34	2
Average number of articles	2.38	2.25	2.28	0.7827	0.68	2
Average num. of categories	1.53	1.46	1.48	0.5563	0.76	2
Average number of visits	1.18	1.43	1.31	3.2093	0.20	2
Average annual expenditure	127.46	141.80	149.45	0.4806	0.79	2
*N*	233	564	403			

## Discussion and conclusions

In accordance with the aim of this research, the outcomes of this study are an improvement over the divergent academic contributions that consider loyalty programs either to act on behavioral loyalty or to be mere promotional tools, with no long-term effect on consumer behavior.

This study shows that, when properly managed, loyalty programs can indeed affect behavioral loyalty, although they are not capable of modifying some of the variables used to measure it. It can thus be concluded that loyalty programs are an ideal tool for managing some of the components comprising customers' behavioral loyalty to commercial establishments.

With respect to the results obtained, as was the case with Lewis (2004), it was confirmed that loyalty programs increased the frequency of visits to the member establishment, thus having a very positive effect on it by: (a) offering greater opportunities for cross-selling; (b) encouraging personal communication and the customization and handling of the offer as opposed to mass-media communication; and (c) diminishing the probability of the customer visiting competing establishments. The company managing the program also benefitted from this increase in the number of visits to one of its member establishments since this, in turn, increased the brand awareness of the program in the minds of its members: acting as a reminder of its possible use in other member establishments and informing them about their accumulated points.

However, joining the program did not have the hoped-for effects on the rest of the buying behavior variables. These results were along the same lines as other previous research that limited the benefits of loyalty programs in terms of loyalty (Dowling and Uncles, 1997; García Gómez et al., 2006) and even in promotional terms (Dorotic et al., 2011).

The existence of differences in the profile of those customers who most changed their buying behavior after joining the program was also demonstrated. Within this group of customers, recency was the determinant variable, as those customers whose time-lapse since their last purchase was greatest were those who most changed their buying behavior. That is to say, these were the customers that reacted most favorably to joining the program. Therefore, integrating itself into the program allowed the joining establishment to awaken those customers described as “sleepers.” Nevertheless, it should be pointed out that these customers were not necessarily the most loyal, but rather simply the most sensitive to the activities of the program.

Another variable that defined the profile of those who most changed their buying behavior was that of the greatest average annual expenditure on the primary product of the business before joining the program. The response to joining together with other products, less relevant to the establishment, produced a lesser variation in buying behavior, thus reflecting the importance of analyzing those customers that buy the products making up the core business of the company.

Apart from this, it was verified that those customers who joined the program in the analyzed establishment were not those who most modified their buying behavior after joining, which once again implied that loyalty programs were effective in activating (or re-activating) customers, but not in making them loyal.

It was also confirmed that the category of products sold, in this case less frequent, medium-priced consumer goods, obtained worse results than other types of products more typical of research focused on loyalty programs (flights, food, etc.). For this reason, the results of this work have provided important business decision-taking information for the different stakeholders involved—the program management and the member companies. This information concerns the type of company that should join the program and the need to simultaneously coordinate program activities with those specific to each member brand in order to improve the results.

### Managerial implications

An important decision to be taken by companies, with regard to the incorporation of a loyalty program into their strategies, is to choose the type of program to which they adhere. For many companies, the decision to join a particular multi-vendor platform is based on management complexity and the higher costs associated with mono-sponsor programs. However, in view of the results of this work, companies wishing to optimize the benefits offered by these programs should assess their limited effects on behavioral loyalty and consider that their advantages will be conditioned by the type of company that is associated with them, as well as the development of a management style that favors the company's own objectives against those of all the other companies promoting the same multi-vendor program.

It is important to point out that the company managing the program should not only include associate companies from different sectors in the program, given that behavior in one sector does not affect behavior in the rest, but it should also, preferably, make sure that the services of these associated establishments are complementary. This would greatly improve effectiveness. Likewise, it has also been shown that the relative importance of each member company within the multi-vendor loyalty program is a determining factor in behavioral loyalty. The driving-force companies of the program will improve the behavioral loyalty of their customers to a greater extent than those companies of lesser importance within the program itself. Thus, the total accumulation rate in the program will only be an interesting segmentation variable for the companies that carry more weight within the program.

With regard to the benefits related to loyalty that have been shown, the increased frequency of visits to related businesses has very positive effects for the management of the establishment, to the extent that:

- greater possibilities for cross-selling are offered, which helps to identify the most suitable products for sale through promotions.
- personalized communication and service, as well as targeted advertising campaigns, is favored in comparison to communications made through mass media.
- the chances that visits will be made to local competitors are diminished.

The fact that multi-vendor programs represent a very useful marketing tool which permits the management of a heterogeneous client base is also verified. From an operational point of view, the ability to identify the profiles of customers who are more sensitive to program actions subsequent to joining, highlights the need to carry out differentiated relationship activities. However, the process of customer segmentation should be made independently by each of the associated companies and not by the company managing the program. This recommendation, which is derived from the results obtained jointly in the program, does not correspond to the behavior seen in each of the participating establishments as previously explained. In this regard, it is recommended that those responsible for the companies that are associated with a multi-vendor program manage information proactively, adapting it to their needs. This implies a greater commercial training of managers and employees in contact with customers, in addition to building a good relationship with the program promoters, which will help the program to adapt, as well as develop specific targets for each establishment based on their situation and the developments in their respective markets. This has obvious implications for companies that choose to adopt multi-vendor programs in order to reduce their marketing costs. For example, a company associated with these programs does not avoid—if they wish to optimize their benefits—having to realize their own communication actions since the generic communication program integrates all associated brands, making it less effective. Communication activities of the program are designed to benefit the whole program and its objectives do not necessarily match the conditions that each of the companies taking part in the program require. Since communications made by the program are not sufficiently segmented, each associated company must decide what their objectives are and what to communicate. Therefore, if an associated company is participating more actively in the management of the program to suit their own particular needs, they will obtain better results, thus improving customer loyalty.

### Limitations and further research

The relationships between loyalty programs and behavioral outcomes are more complex than has been assumed. This empirical investigation had certain limitations: the effect of the loyalty program was only tested with regard to behavioral loyalty. The integration of attitudinal variables to complement the behavioral approach to loyalty would be a promising area of research.

It would likewise be interesting to continue to progress with the exploratory models used in this study, with a view to more precisely explaining changes in buying behavior after a customer joins a multi-sponsor loyalty program. Therefore, new variables should be incorporated related to purchases at competing establishments, such as share of wallet, a variable that, as seen in the literature review, has been included in numerous studies on behavioral loyalty. In other words, it should be determined whether the increase in the number of store visits is concentrated in the program and, therefore, decreases the number of visits to the competition or if, on the contrary, the frequency of visits increases under the program but remains unchanged with regard to the rest of the stores. This would mean that, although the program would improve purchasing behavior at the associated establishment, high levels of customer loyalty would not be achieved, as customers would continue to divide their purchases between the different competing brands.

Additionally, future studies should address segmentation in loyalty programs based on customer lifetime value. As proposed by Kumar and Shah (2004), it would be interesting to measure customers' patronage behavior, taking into account the expected future value and not just past data.

Finally, given that the data analyzed correspond to a single multi-sponsor loyalty program (as in other studies conducted at the international level), and even though the program's representativeness is justified as there is only one other program with the same characteristics in Spain, it would be advisable to compare the results with those of other international programs.

## Author contributions

The directors of research have been the professors PR and ER. The three co-authors have participated in all stages of work, including the conception and design of the research, the revision of intellectual content, and drafting the work. The analysis and interpretation of data has been the responsibility of the professor TV.

### Conflict of interest statement

The authors declare that the research was conducted in the absence of any commercial or financial relationships that could be construed as a potential conflict of interest.
